# Akt/PKB Controls the Activity-Dependent Bulk Endocytosis of Synaptic Vesicles

**DOI:** 10.1111/j.1600-0854.2012.01365.x

**Published:** 2012-04-29

**Authors:** Karen J Smillie, Michael A Cousin

**Affiliations:** Membrane Biology Group, Centre for Integrative Physiology, George Square, University of EdinburghEdinburgh, EH8 9XD, UK

**Keywords:** Akt, dynamin, endocytosis, fluid phase, glycogen synthase kinase 3, presynapse, vesicle

## Abstract

Activity-dependent bulk endocytosis (ADBE) is the dominant SV endocytosis mode during intense neuronal activity. The dephosphorylation of Ser774 on dynamin I is essential for triggering of ADBE, as is its subsequent rephosphorylation by glycogen synthase kinase 3 (GSK3). We show that in primary cultures of cerebellar granule neurons the protein kinase Akt phosphorylates GSK3 during intense neuronal activity, ensuring that GSK3 is inactive during intense stimulation to aid dynamin I dephosphorylation. Furthermore, when a constitutively active form of Akt was overexpressed in primary neuronal cultures, ADBE was inhibited with no effect on clathrin-mediated endocytosis. Thus Akt has two major regulatory roles (i) to ensure efficient dynamin I dephosphorylation via acute activity-dependent inhibition of GSK3 and (ii) to negatively regulate ADBE when activated in the longer term. This is the first demonstration of a role for Akt in SV recycling and suggests a key role for this protein kinase in modulating synaptic strength during elevated neuronal activity.

The maintenance of neurotransmission at central nerve terminals is dependent on the efficient retrieval and recycling of synaptic vesicles (SVs) across a wide range of stimuli. During mild synaptic activity the dominant endocytosis mode is clathrin-mediated endocytosis (CME), which retrieves single SVs from the nerve terminal membrane (1,2). However, when neuronal activity increases, an additional endocytosis mode is triggered to provide a rapid and immediate increase in SV retrieval capacity, called activity-dependent bulk endocytosis (ADBE (3). ADBE immediately corrects for gross changes in nerve terminal surface area via the rapid generation of endosomes direct from the plasma membrane. SVs can then bud from these endosomes to rejoin the SV reserve pool (4,5).

ADBE is triggered by the activity-dependent dephosphorylation of the large GTPase dynamin I on two specific sites (Ser774 and Ser778) by the calcium-dependent protein phosphatase calcineurin (6). This dephosphorylation permits an interaction with syndapin I (7), a protein also essential for ADBE (6). After stimulation dynamin I is rephosphorylated by cyclin-dependent kinase 5 (cdk5) on Ser778, which primes Ser774 for phosphorylation by glycogen synthase kinase 3 (GSK3 (8). The activities of both cdk5 and GSK3 are essential for maintaining subsequent rounds of ADBE (8,9) indicating dynamin I rephosphorylation is equally important as its dephosphorylation.

GSK3 activity is inhibited by its phosphorylation by several different protein kinases (10), the best characterized GSK3 kinase being Akt (Protein kinase B) (11,12). Akt is a serine/threonine kinase with three isoforms: the ubiquitously expressed Akt 1 and 2, and Akt 3 which is primarily expressed in the brain and testis (12). Akt is activated by its phosphorylation on two major sites (Thr308, Ser473) by upstream signalling cascades including the phosphatidylinositol-dependent kinase 1 (PDK1) and mTor/rictor pathways (13,14).

Since GSK3 has a high basal level of activity (10), we hypothesized that it may be inhibited during intense neuronal activity, to ensure dynamin I is maximally dephosphorylated. We found that GSK3 was phosphorylated (and thus inactivated) by Akt only during high intensity stimulation, identifying Akt as an activity-dependent GSK3 kinase. As predicted, inhibition of Akt resulted in reduced dephosphorylation of dynamin I during strong stimulation. Further experiments using overexpression of constitutively active Akt revealed that it is also a negative regulator of ADBE, while having no role in CME-dependent SV turnover. Thus, Akt controls ADBE via regulation of presynaptic GSK3 activity, which is the first demonstration of a role for Akt in the regulation of SV recycling in central nerve terminals.

## Results

### Akt inhibits GSK3 in an activity-dependent manner

**Figure 1:**
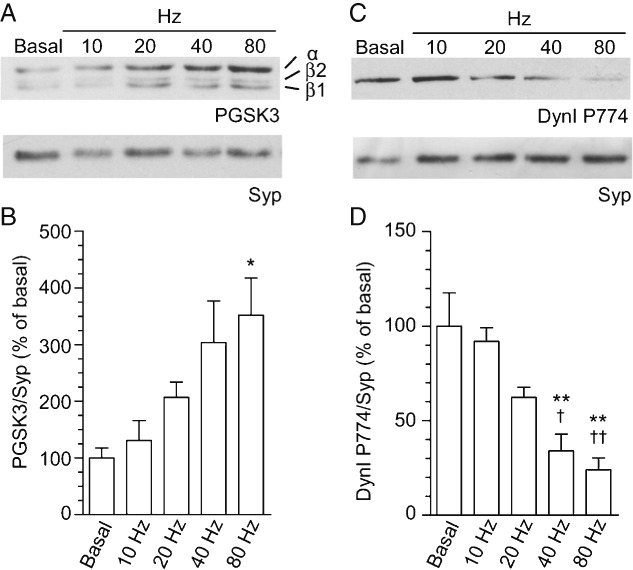
GSK3 is phosphorylated in an activity-dependent manner Cultures were subjected to action potential trains of increasing frequency (10, 20, 40 or 80 Hz) for 10 seconds. The extent of phosphorylation of either Ser9/21 on GSK3β/α (PGSK3 (A) or Ser774 on dynamin I (DynI P774 (C) was assessed by western blotting. Synaptophysin (Syp) blots were performed as loading controls. Representative blots are displayed for all experiments, with the three isoforms of GSK3 (α, β1 and β2) indicated in (A). The extent of phosphorylation of either GSK3 Ser9/21 (B) or dynamin I Ser774 (D) is displayed. Data were corrected against protein levels (Syp) and then normalized to basal ± SEM (*n* = 4 for both GSK3 and dynamin). One-way anova: *p < 0.05, **p < 0.01 to basal, ^†^p < 0.05, ^††^p < 0.01 to 10 Hz.

The activity-dependent dephosphorylation of Ser774 on dynamin I by calcineurin is essential for ADBE (6) as is its subsequent rephosphorylation by GSK3 (8). Since GSK3 has a high basal activity, we hypothesized that it may be inactivated during high intensity stimulation to ensure efficient dynamin I dephosphorylation. To test this hypothesis, we monitored GSK3 activity in primary neuronal cultures across a range of different stimulation intensities. GSK3 activity was determined by probing the phosphorylation status of Ser9/Ser21 of GSK3β/α, since phosphorylation on this site inhibits the enzyme (10,11). We observed a dramatic activity-dependent increase in GSK3 phosphorylation, ranging from no effect of low intensity stimulation (10 Hz) to maximal phosphorylation during high stimulation intensity (Figure 1A,B). Thus, GSK3 is phosphorylated and inhibited in an activity-dependent manner. A reciprocal activity-dependent dephosphorylation of dynamin I was observed under identical conditions (Figure 1C,D) (6). Thus during mild stimulation (where CME is dominant) GSK3 is active and calcineurin is inactive, resulting in maintenance of Ser774 phosphorylation on dynamin I. However during intense stimulation, GSK3 is inhibited and calcineurin is activated, which should allow efficient dephosphorylation of Ser774 on dynamin I.

**Figure 2:**
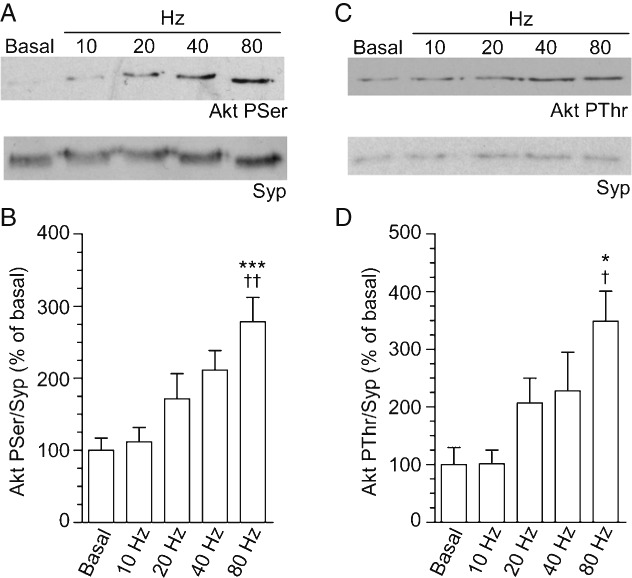
Akt is phosphorylated in an activity-dependent manner Cultures were subjected to action potential trains of increasing frequency (10, 20, 40 or 80 Hz) for 10 seconds. The extent of phosphorylation of either Akt Ser473 [Pser, (A)] or Thr308 [PThr (C)] was assessed by western blotting. Synaptophysin (Syp) blots were performed as loading controls. Representative blots are displayed for all experiments. The extent of phosphorylation of either Akt Ser473 (B) or Thr308 (D) is displayed. Data were corrected against protein levels (Syp) and then normalized to basal ± SEM (*n* = 7 for PSer Akt and *n* = 5 for PThr Akt). One-way anova: *p < 0.05, ***p < 0.001 to basal; ^†^p < 0.05, ^††^p *<* 0.01 to 10 Hz.

We next investigated which protein kinase was responsible for the activity-dependent phosphorylation of GSK3. A prime candidate is Akt, which is the best characterized GSK3 kinase (11,12). Akt is activated when phosphorylated, therefore as a first step we determined whether Akt phosphorylation followed the same stimulation-dependent pattern to that observed with GSK3, by western blotting with phospho-specific antibodies against both Ser473 and Thr308. Low intensity stimulation had no effect on the phosphorylation status of either residue, whereas the phosphorylation of both residues scaled with increasing stimulation intensity (Figure 2). Thus activation of Akt follows an identical pattern to the inactivation of GSK3, suggesting that Akt is the activity-dependent GSK3 kinase in central nerve terminals.

**Figure 3:**
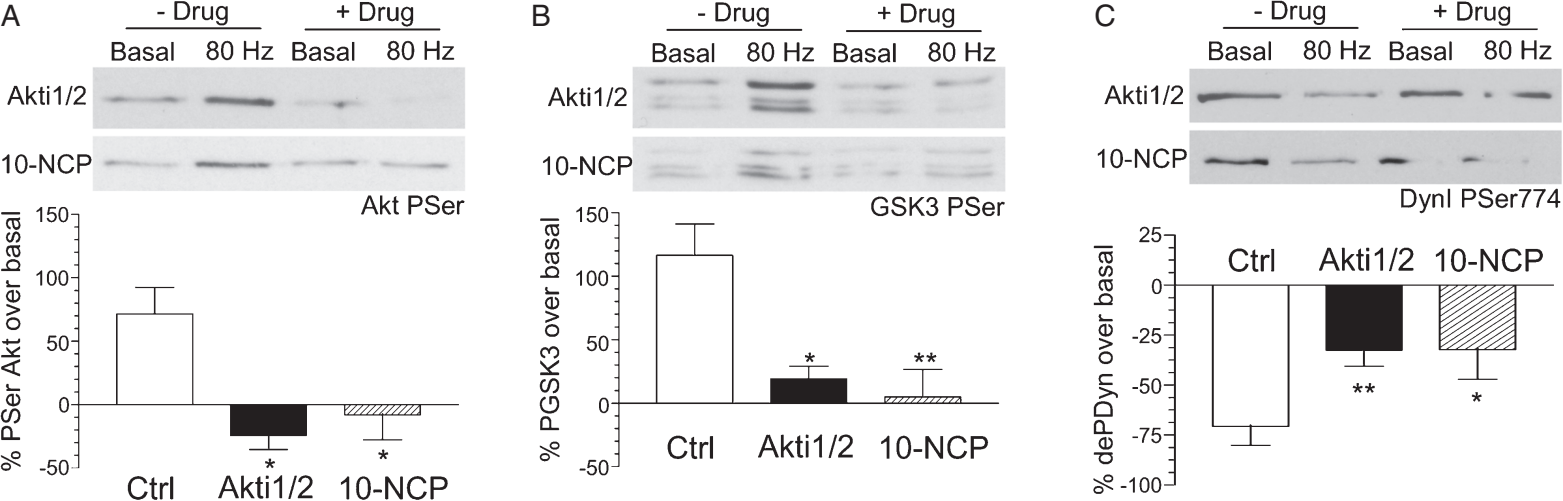
Akt phosphorylates GSK3 to retard dynamin I dephosphorylation during high intensity stimulation Cultures were incubated either in the absence or presence of Akt antagonists (Akti1/2–500 nm, 10-NCP–500 nM) for 10 min. Cultures were then either stimulated (80 Hz 10 seconds) or rested (basal) for 10 seconds in the absence and presence of antagonists and then immediately lysed. Representative blots display the phosphorylation of either (A) Akt Ser473, (B) GSK3 Ser9/21 or (C) dynamin I (DynI) Ser774 in the absence (− Drug) or presence (+ Drug) of either Akti1/2 or 10-NCP. The extent of phosphorylation/dephosphorylation of either Akt (A), GSK3 (B) or DynI (C) in the absence of inhibitor (Ctrl, clear bars), the presence of Akti1/2 (filled bars) or 10-NCP (hatched bars) is displayed. Data were corrected against protein levels (Syp) and expressed as the extent of stimulus-evoked phosphorylation over basal ± SEM (*n* = 8 for PAkt control, *n* = 3 for PAkt Akti1/2, *n* = 3 for PAkt 10-NCP; *n* = 8 for PGSK3 control, *n* = 5 for PGSK3 Akti1/2, *n* = 5 for PGSK3 10-NCP; *n* = 17 for PDynI control, *n* = 13 for PDynI Akti1/2, *n* = 6 for PDynI 10-NCP). Student's *t*-test: *p < 0.05, **p < 0.01.

To confirm Akt as the activity-dependent GSK3 kinase, cultures were incubated with two independent Akt antagonists. Akti1/2 inhibits Akt phosphorylation by preventing access to an activation loop that is revealed on plekstrin homology (PH) domain binding to lipid (15), whereas 10-NCP is thought to compete for ATP binding to the enzyme (16). Exposure to either Akt antagonist abolished Akt phosphorylation evoked by high intensity stimulation as expected (Figure 3A). Importantly, both antagonists also abolished high-intensity stimulation-evoked GSK3 phosphorylation under identical experimental conditions (Figure 3B). Thus, Akt is the activity-dependent GSK3 kinase in central nerve terminals.

**Figure 4:**
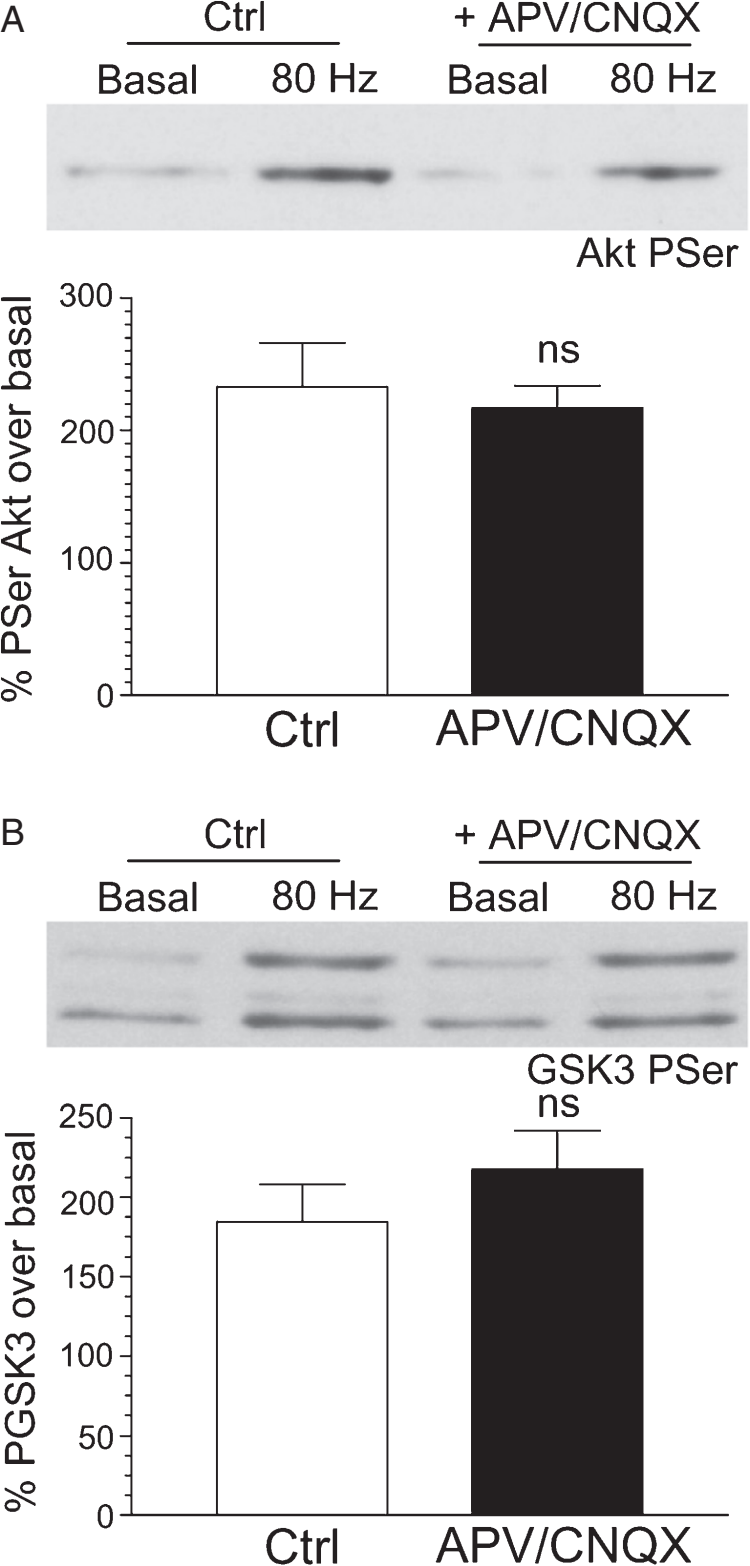
Activity-dependent phosphorylation of both Akt and GSK3 is presynaptic Cultures were incubated either in the absence or combined presence of ionotropic glutamate receptor antagonists (APV, 50 µm, CNQX, 10 µm) for 10 min. Cultures were then either stimulated (80 Hz 10 seconds) or rested (Basal) for 10 seconds in the absence and presence of antagonists and then immediately lysed. Representative blots display the phosphorylation of either (A) Akt Ser473 (Akt PSer) or (B) GSK3 Ser9/21 (GSK3 PSer) in the absence (Ctrl) or presence (+ APV/CNQX) of antagonists. The extent of phosphorylation of either Akt (A) or GSK3 (B) in the absence of inhibitor (Ctrl, clear bars) or the presence of APV/CNQX (filled bars) is displayed. Data were corrected against protein levels (Syp) and expressed as the extent of stimulus-evoked phosphorylation over basal ± SEM (*n* = 4 for PAkt Ctrl and APV/CNQX, *n* = 12 for PGSK3 Ctrl and APV/CNQX). Student's *t*-test: ns = not significant.

Both Akt and GSK3 have key roles in postsynaptic function, including control of synaptic strength and plasticity via AMPA receptor trafficking (17–19), with the phosphorylation of Akt postulated to be downstream from activation of ionotropic glutamate receptors (20,21). Therefore, the activity-dependent phosphorylation of Akt and GSK3 observed in our cultures may be a result of postsynaptic, rather than presynaptic changes. To determine this, cultures were incubated with a cocktail of ionotropic glutamate receptor antagonists and then challenged with a train of 800 action potentials (80 Hz). The activity-dependent phosphorylation of both Akt and GSK3 was unaffected by inhibition of ionotropic glutamate receptors confirming that these events were presynaptic, and not postsynaptic (Figure 4).

### Akt permits efficient dephosphorylation of dynamin I during intense stimulation

To test whether activity-dependent inhibition of GSK3 by Akt permit the efficient dephosphorylation of dynamin I, we next determined the effect of inhibiting Akt on this event. In control cultures dynamin I was robustly dephosphorylated on Ser774 by a train of 800 action potentials (80 Hz, Figure 3C). However, in the presence of either Akti1/2 or 10-NCP the extent of dephosphorylation was significantly reduced by approximately 50% (Figure 3C). Thus, the activation of Akt ensures maximal dephosphorylation of dynamin I on Ser774 by transiently inhibiting GSK3 during strong stimulation.

**Figure 5:**
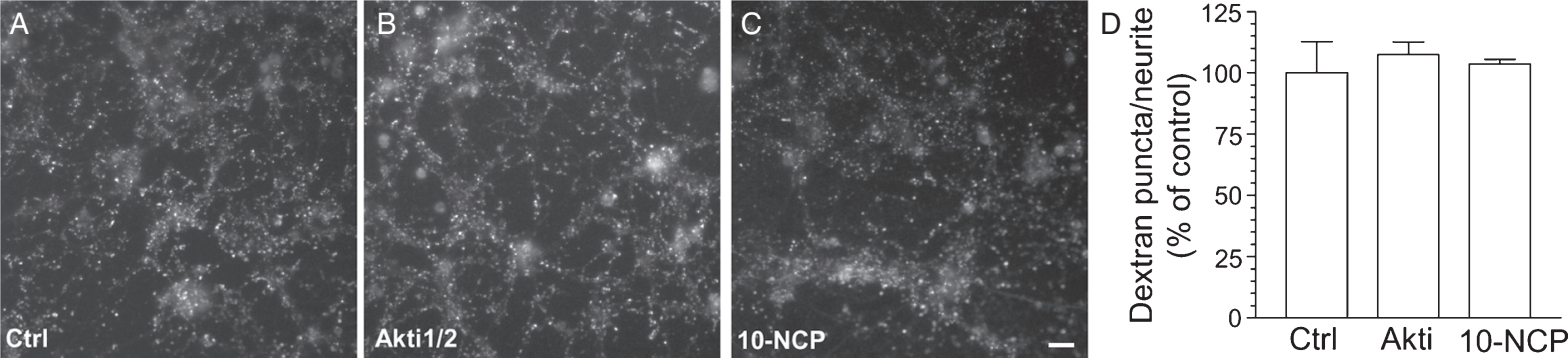
Effect of acute Akt inhibition on the uptake of large dextrans Cultures were incubated either in the absence or presence of Akt antagonists (Akti1/2, 500 nm, 10-NCP, 500 nm) for 10 min. Cultures were then stimulated (80 Hz 10 seconds) in the presence of 50 μm tetramethylrhodamine-dextran followed by immediate washout. A–C) Panels show representative fields of view of dextran loading in either control (Ctrl, A), or cultures treated with either Akti1/2 (B) or 10-NCP (C). Scale bar represents 20 µm. (D) Quantification of the extent of dextran uptake (puncta number per field) expressed as a percentage of control neurons ± SEM (*n* = 3 independent experiments for all). One-way anova performed, all not significant.

Dephosphorylation of Ser774 is essential for triggering ADBE (6,8). Since inhibition of Akt significantly reduced Ser774 dephosphorylation, we next addressed whether the extent of ADBE was reduced in a parallel fashion. ADBE was quantified by monitoring uptake of a large fluorescent dextran (tetramethlyrhodamine-dextran, 40 kDa) that is too large to be accumulated into single SVs (22). A train of 800 action potentials resulted in a robust uptake of dextran in control conditions (Figure 5A,D). Inhibition of Akt with either Akti1/2 or 10-NCP resulted in a similar amount of uptake compared to control (Figure 5B–D). Therefore, even though dynamin I dephosphorylation is significantly blunted by inhibition of Akt, it is still sufficient to trigger ADBE.

### Akt negatively controls ADBE but has no role in CME

**Figure 6:**
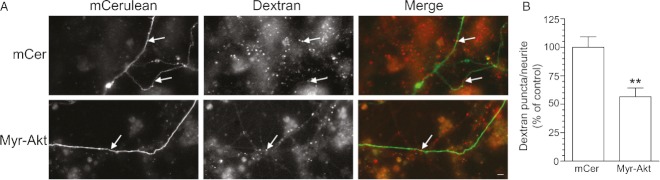
Constitutively active Akt inhibits the uptake of large dextrans Cultures were transfected with either mCerulean alone or co-transfected with mCerulean and myristoylated-Akt (myr-Akt) 48 h prior to imaging. Tetramethylrhodamine-dextran (50 µm) was loaded with action potential stimulation (80 Hz 10 seconds) followed by immediate washout. A) Left panels show transfected neurites (mCer) with either mCerulean alone (mCer) or co-expressed with myr-Akt. Middle panels show dextran uptake. Right panels show the merged image with the transfected neurite in green and dextran uptake in red. Arrows indicate points of dextran loading. Scale bar represents 10 µm. B) Quantification of the extent of dextran uptake (puncta number) in myr-Akt neurites, expressed as a percentage of neurites transfected with empty mCerulean vector (mCer) ± SEM (*n* = 10 mCer; *n* = 9 myr-Akt). Student's *t*-test: **p < 0.01.

The acute activity-dependent inhibition of GSK3 by Akt did not sufficiently retard dynamin I dephosphorylation to impact on the extent of ADBE. However, longer term activation of Akt may result in effective negative regulation of ADBE, since the constitutive activity of GSK3 is essential for the maintenance of this endocytosis mode (8). To test this, a constitutively active form of the enzyme, myristoylated-Akt (myr-Akt) (23) was overexpressed in our cultures and the extent of ADBE was quantified by monitoring uptake of dextran. Robust dextran uptake was observed in cultures transfected with a control fluorescent vector (mCerulean) in response to high intensity stimulation (800 action potentials at 80 Hz, Figure 6). In contrast, neurons transfected with myr-Akt displayed a significant reduction in dextran uptake compared to mCerulean-transfected controls (Figure 6). Thus Akt is a negative regulator of ADBE in central nerve terminals when activated in the longer term.

**Figure 7:**
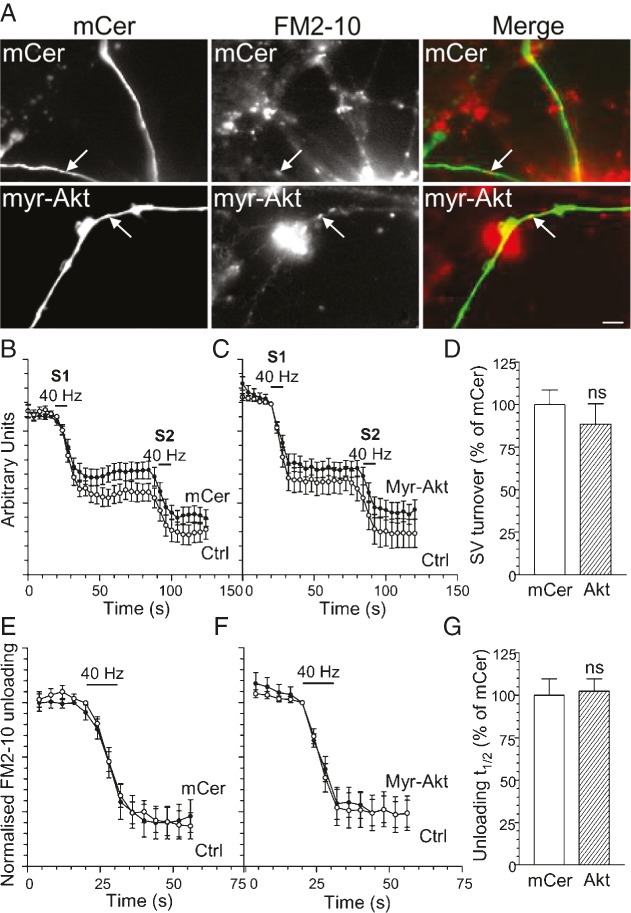
Overexpression of constitutively active Akt does not affect CME or SV exocytosis SV turnover was assayed in neurons transfected with either mCerulean (mCer) alone or co-transfected with mCer and myristolated-Akt (myr-Akt) 48 h prior to imaging. FM2-10 (100 µm) was loaded with action potential stimulation (80 Hz 10 seconds) followed by immediate washout. After 10 min, the internalized dye was unloaded using two sequential 400 action potential stimuli (40 Hz, S1 and S2). A) Left panels show transfected neurites (mCer) with either mCerulean alone or co-expressed with myr-Akt. Middle panels show FM2-10 uptake. Right panels show the merged image with the transfected neurite in green and FM2-10 in red. Arrows indicate points of FM2-10 loading. Scale bar represents 15 µm. B and C) Examples of average unloading profiles of transfected neurites (closed circles) and untransfected neurites (open circles) in the same field of view ± SEM (B, mCer; C, myr-Akt). D) Average SV turnover normalized to neurons expressing mCerulean ± SEM. E and F) The evoked decrease in FM2-10 fluorescence from S1 was normalized between 1 and 0 to quantify unloading kinetics. Time course of dye unloading from transfected nerve terminals (closed circles) and untransfected nerve terminals (open circles) in the same field of view are displayed (E, mCer; F, myr-Akt) ± SEM. G) The average time taken to lose 50% of dye (*t*_1/2_) is displayed normalized to neurons expressing mCerulean ± SEM. In all experiments *n* = 4; Student's *t*-test: ns = not significant.

We next determined whether Akt activity selectively regulated ADBE or whether it also controlled CME. To test this we monitored SV turnover using the fluorescent dye FM2-10, which only labels SVs retrieving via CME (6). Transfected cultures were loaded with dye using 800 action potentials (80 Hz), left to recover and then maximally unloaded with two further stimuli (400 action potentials at 40 Hz). The extent of dye unloading is indicative of the number of SVs retrieved and recycled by CME (SV turnover). The extent of SV turnover in myr-Akt transfected neurons was not significantly different to those expressing empty mCerulean vector (Figure 7B–D). In addition, overexpression of myr-Akt had no effect on SV exocytosis, since the kinetics of dye unloading (*t*_1/2_) were not significantly different to control mCerulean transfected neurons (Figure 7E–G). Thus, Akt activity has no role in either CME-mediated SV recycling or SV exocytosis, highlighting an exclusive role as a negative regulator of ADBE.

## Discussion

We have shown that Akt regulates ADBE via its inhibition of presynaptic GSK3. Akt transiently inhibits GSK3 during elevated, but not low, neuronal activity, resulting in the efficient and maximal dephosphorylation of dynamin I by calcineurin. The inhibition of GSK3 by Akt results in a negative regulation of ADBE when Akt is activated for longer time periods. Thus, Akt facilitates dynamin I dephosphorylation during strong stimulation, but retards its rephosphorylation by GSK3 when constitutively activated. This is the first demonstration of a role for Akt in SV recycling and suggests that signalling cascades that modulate Akt activity will have selective and specific inhibitory effects on ADBE.

We have uncovered a novel role for Akt in SV recycling in central nerve terminals via its downstream phosphorylation of GSK3. Activation of presynaptic Akt was visualized using phospho-specific antibodies against two key sites (Ser473 and Thr308). The nature of our experiments (whole sample used for analysis) did not allow normalization against total Akt using pan-Akt antibodies. Instead we normalized protein levels by reprobing our blots with antibodies against the SV protein synaptophysin. Importantly, we confirmed that pan-Akt and synaptophysin levels co-varied across a range of different samples (K. J. S. data not shown).

Previous studies have highlighted a role for Akt in the trafficking and fusion of secretory vesicles, the best characterized of which is in the trafficking of the glucose transporter GLUT4 in muscle cells. In this system insulin-dependent activation of Akt stimulates the redistribution of GLUT4 transporters to the plasma membrane via an Akt-dependent phosphorylation of Akt substrate 160 (reviewed in (24). Akt is also required for the translocation and insertion of both glutamate transporters in glioma cells (25) and GABA_A_ receptors at the postsynapse (26). The latter event resulted in increased synaptic strength via a direct phosphorylation of the GABA_A_ receptor by Akt in response to insulin. Akt can also regulate the docking or fusion of vesicles in several secretory cells (27–30). However, our work highlights the absence of a role for Akt in SV exocytosis at the presynapse, with overexpression of constitutively active Akt leaving both SV turnover and release kinetics unaltered.

The phosphorylation of both postsynaptic Akt and GSK3 during either chronic depolarization or prolonged synaptic activity in culture is well documented, with Akt phosphorylation essential for neuronal survival (20,21,31–33). This essential role precluded the use of dominant negative forms of Akt in our studies, since their overexpression resulted in neuronal death ( (31), M. A. C. unpublished observations). However, the acute and rapid activation of Akt observed during trains of high frequency action potentials suggests an additional presynaptic role for the enzyme that is discrete from its essential role in neuronal survival. The presynaptic activation of Akt was confirmed in experiments that showed comparable action potential-evoked Akt phosphorylation in the absence or presence of ionotropic glutamate receptor antagonists. How could increased neuronal activity be transduced into activation of presynaptic Akt? A potential route for activity-dependent Akt phosphorylation is the calcium-dependent activation of Ras by Ras-GRF exchange factor (34). In support, overexpression of dominant negative Ras inhibits Akt phosphorylation evoked by elevated KCl in sympathetic neurons (32). Antagonists of phosphatidylinositol 3-kinase (PI3K) reversed this KCl-evoked increase in Akt phosphorylation, suggesting an involvement of PDK1/PI3K downstream of the Ras signalling pathway (32). Interestingly, Akt phosphorylation during chronic KCl exposure is not dependent on PI3K (20), suggesting acute and chronic membrane depolarization may couple to different signalling cascades to phosphorylate Akt, or alternatively activation of Akt in different presynaptic and postsynaptic compartments. Another possible mechanism that couples acute neuronal activity to Akt activation is the direct activation of PI3K by calmodulin (35). This is potentially the most intriguing because calcineurin activation is dependent on both calcium influx and calmodulin (36). If calmodulin were to also activate Akt via PI3K, then calmodulin would be revealed as a key modulator of dynamin I dephosphorylation, via the simultaneous activation of calcineurin and inhibition of GSK3. The molecular identity of the cascade that mediates activity-dependent Akt phosphorylation is the subject of current investigation in our laboratory.

The acute activity-dependent inhibition of GSK3 by Akt resulted in a significant reduction in the extent of dynamin I dephosphorylation by calcineurin. This reduction was not sufficient to affect ADBE, suggesting that a threshold level of dynamin I dephosphorylation exists that allows maximal triggering of this endocytosis mode. It is unknown how many dephosphorylated dynamin I molecules are sufficient to trigger ADBE; however, a decrease of only 30% of the total phosphorylated pool is sufficient to maximally trigger ADBE (6,22). This agrees with data presented here, where Akt inhibitors retarded global dynamin I dephosphorylation to approximately 30% with no effect on triggering of ADBE. Alternatively, rather than a global dephosphorylation event, a small pool of dephosphorylated dynamin I in a specific subcellular localization may be critical to trigger ADBE. Regardless, the activity-dependent inhibition of GSK3 by Akt can be viewed as a ‘fail-safe’ mechanism to ensure that dynamin I dephosphorylation always exceeds the triggering threshold for ADBE during intense stimulation.

The inhibition of ADBE by constitutively active Akt suggests that this enzyme may be a key control point for negative regulation of this endocytosis mode if activated in the longer term. While constitutively active Akt is used as a research tool in this study purely to demonstrate a molecular role for Akt in ADBE, long-term activation of Akt can occur in a number of pathological and physiological contexts. For example, rats subjected to intermittent normobaric hyperoxia after transient focal ischaemia displayed prolonged activation of Akt for up to 24 h (37). A more conventional mechanism for longer term activation of Akt is through signalling cascades. Many different cascades converge on Akt (12), however, the neurotrophin class of signalling molecules are of particular interest, because they control synaptic function and plasticity in mature synapses (38). For example, brain-derived neurotrophic factor is differentially released from dendrites and nerve terminals dependent on neuronal activity (39). Preliminary experiments in our laboratory have confirmed that Akt is activated on exposure to brain-derived neurotrophic factor (unpublished observations). Thus, specific trains of stimuli may evoke the localized release of signalling molecules that impact on the extent of ADBE in neighbouring neurons. This would provide a novel mechanism to regulate local synaptic strength during intense neuronal activity. We have recently found that ADBE is triggered by similar stimulation intensities in cultures of hippocampal neurones (M. A. C., unpublished observations), suggesting Akt-dependent control of this endocytosis mode may be prevalent across many different brain regions. In support, inhibition of presynaptic GSK3 translates into a relief of short-term synaptic depression of hippocampal neurotransmission during high intensity stimulation (8).

We have shown a direct role for Akt in SV recycling in central nerve terminals for the first time. During intense stimulation, the activity-dependent phosphorylation of Akt inhibits GSK3, ensuring maximal dephosphorylation of dynamin I. However when Akt is continually activated, it inhibited ADBE by preventing GSK3-dependent rephosphorylation of dynamin I. This regulation will be of critical importance, because ADBE is the major SV endocytosis mode that should be active during events such as long-term potentiation or pathological conditions such as epileptic discharge. Thus modulators of Akt signalling may have the potential to alter cognitive ability and possibly suppress seizure activity. The next challenge is to identify these signalling routes and determine their role in activity-dependent SV recycling in central nerve terminals.

## Materials and Methods

### Materials

FM2-10, tetramethylrhodamine-dextran, penicillin/streptomycin, phosphate-buffered salts and minimal essential medium were obtained from Invitrogen. Foetal calf serum was from Biosera. The dynamin I phosphospecific Ser774 antibody was from Abcam, the synaptophysin antibody was from Synaptic Systems, the phospho-Akt Ser473, phospho-Akt Thr308 and phospho-GSK3 β/α Ser9/21 antibodies were from Cell Signalling. Akti1/2 was a gift from Dr. Calum Sutherland (University of Dundee), 10-NCP (10-[4^′^-(*N*-diethylamino)butyl]-2-chlorophenoxazine, Akt Inhibitor X) was from Calbiochem while 6-cyano-7-nitroquinoxaline-2,3-dione (CNQX) and 2-amino-5-phosphopentanoic acid (APV) were from Tocris. The myristoylated-Akt vector (myr-Akt) was a gift from Prof. Alan Morgan (University of Liverpool). All other reagents were from Sigma.

### Primary cell culture and transfections

Primary cultures of cerebellar granule neurons (CGNs) were prepared from the cerebella of 7-day old Sprague-Dawley rat pups (40). CGNs were transfected between 6 and 7 days *in vitro* using calcium phosphate precipitation (40) and imaged 48 h later. For all experiments, cultures were removed from culture medium into incubation medium [in mm: 170 NaCl, 3.5 KCl, 0.4 KH_2_PO_4_, 20 TES (*N*-tris[hydroxy-methyl]-methyl-2-aminoethane-sulfonic acid), 5 NaHCO_3_, 5 glucose, 1.2 Na_2_SO_4_, 1.2 MgCl_2_, 1.3 CaCl_2_, pH 7.4] for 10 min before commencing experiments.

### Assays of protein phosphorylation status

Cultures were stimulated with trains of action potentials of differing frequency (10–80 Hz) for 10 seconds. Immediately after stimulation, SDS sample buffer (67 mm SDS, 2 mm EGTA, 9.3% glycerol, 12% β-mercaptoethanol, bromophenol blue, 67 mm Tris, pH 7.4) was added to lyse the neurons. Lysate was quickly removed and boiled for subsequent analysis by SDS-PAGE and western blotting. In experiments with Akt antagonists, drugs were included in incubation medium before (10 min) and during stimulation. Primary antibodies were all used at a dilution of 1:1000. The intensity of the signal from the phospho-dynamin, phospho-GSK3 and phospho-Akt blots was normalized against the amount of synaptophysin and expressed either as a percentage of control or as a percentage of evoked phosphorylation.

### Fluorescence imaging of dextran uptake and SV turnover

Fluorescent images were captured using a Hamamatsu Orca-ER CCD digital camera attached to a Zeiss Axio Observer D1 epifluorescence microscope with either a 20× air or 40× oil objective. Images were processed using offline imaging software (Image J). The uptake of tetramethylrhodamine-dextran (40 kDa) was monitored as described (22). Briefly, cultures were stimulated with a train of 800 action potentials (80 Hz) in the presence of 50 µm tetramethylrhodamine-dextran. Non-internalized dextran was then immediately washed away by perfusion. Dextran uptake was visualized at 550 nm excitation, with emission fluorescence gathered at >575 nm. The extent of dextran uptake was determined by the number of fluorescent puncta per field of view for the Akt inhibitor experiments (at least 10 fields of view per experiment) and per transfected neuron (at least three neurons per coverslip).

The imaging of SV turnover was monitored as described (6). Briefly, SV turnover was initiated with a train of 800 action potentials (80 Hz) and invaginating membrane was loaded with FM2-10 (100 µm). Dye was washed from the cultures immediately after stimulation. After a 10 min rest period, accumulated dye was unloaded from nerve terminals with two sequential 400 action potential (40 Hz) stimuli, 1 min apart. Transfected neurons were visualized at 430 nm excitation and FM2-10 was visualized at 500 nm (both >525 nm emission). SV turnover in transfected neurons was calculated as the total dye released during stimulation (ΔS1 + ΔS2) as a percentage of untransfected neurons in the same field of view. Dye unloading kinetics were analyzed by normalizing the S1 fluorescence unload between 0 and 1 and calculating the time taken for 50% of the dye to be released (*t*_1/2_). A minimum of 10 nerve terminals per neurite were analyzed during each experiment.
